# D1 Receptor Agonist Ameliorates Synaptic and Behavioral Deficits in a *Shank3*‐Deficient Mouse Model of Autism

**DOI:** 10.1002/mco2.70428

**Published:** 2025-10-15

**Authors:** Jun‐Sik Kim, Sukmin Han, Mihyeon An, Jinsu Park, Yeongyeong Lee, Jeein Lim, Sung Hyun Kim, Dong‐Gyu Jo

**Affiliations:** ^1^ School of Pharmacy, Sungkyunkwan University Suwon South Korea; ^2^ Department of Neuroscience Graduate School, Kyung Hee University Seoul South Korea

1

Dear Editor

Autism spectrum disorder (ASD) has been associated with mutations in *SHANK* genes, which encode critical scaffolding proteins at glutamatergic synapses [1]. However, the mechanisms by which *SHANK3* deficiency disrupts dopamine‐dependent cortico‐striatal signaling remain poorly understood. In this study, we revealed that loss of *Shank3* impairs D1 receptor–mediated cortico‐striatal projections, giving rise to ASD‐like behavioral phenotypes. Notably, pharmacological activation of the D1 receptor with SKF‐82958 restored synaptic function and ameliorated repetitive and social behavioral deficits in *Shank3*‐deficient mice. These findings highlight D1 receptor agonism as a potential therapeutic approach for the symptomatic treatment of ASD.

SHANK proteins (SHANK1, SHANK2, and SHANK3) are critical components of the postsynaptic density (PSD) and form a key part of the PSD95/SAPAP/SHANK complex that supports glutamatergic synaptic signaling [1]. Especially, SHANK3 is highly expressed in the striatum, where its deficiency is associated with significant synaptic dysfunction. In *Shank3* −/− mice, reduced expression of NMDA and AMPA receptor subunits in the striatum leads to decreased cortico‐striatal synaptic transmission in striatal medium spiny neurons (MSNs) [1]. MSNs integrate glutamatergic input from the prefrontal cortex with dopaminergic signals from the substantia nigra. D1 receptor‐mediated Gs signaling activates D1 MSNs, whereas D2 receptor‐mediated Gi signaling inhibits D2 MSNs.S1

Here, we used *Shank3B*‐deficient mice as a model for *Shank3* −/− [1]. In the cortex, we observed no significant changes in PSD95 or the cortico‐striatal projection marker vGLUT1. However, the striatum exhibited marked dysregulation of PSD95 and vGLUT1, along with downregulation of dopamine signaling components, particularly DRD1 and DAT (Figure 1A). Based on these findings, we hypothesized that the reduction in D1 receptor expression leads to diminished D1 receptor‐mediated signaling. To restore this signaling pathway, we administered the D1 receptor agonist SKF‐82958. In the marble burying test, which reflects repetitive behaviors, *Shank3* −/− mice buried more marbles than *Shank3* +/+, but this behavior was recovered after SKF‐82958 treatment. Moreover, in the three‐chamber social interaction tests, *Shank3* −/− mice exhibited impaired social interactions, which were ameliorated following SKF‐82958 administration (Figure 1B). These findings collectively indicate that the activation of D1 receptor signaling by SKF‐82958 can reverse ASD‐like behaviors in *Shank3* −/− mice.

**FIGURE 1:**
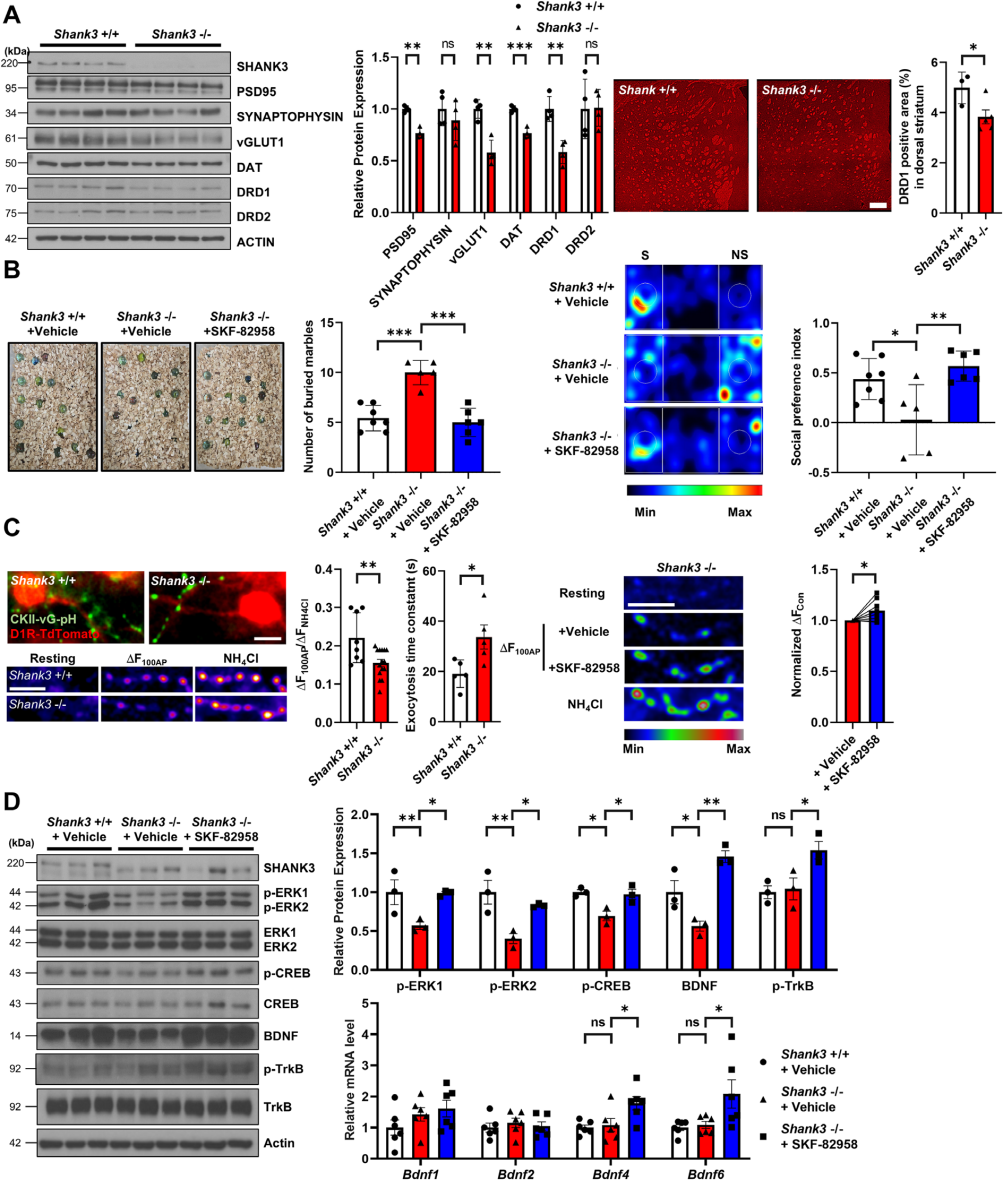
Dysregulation of D1 receptor‐mediated dopamine signaling in the striatum of *Shank3*‐deficient mice is alleviated by the D1 receptor agonist SKF‐82958. (A) Western blot analysis and quantification of SHANK3, PSD95, SYNAPTOPHYSIN, vGLUT1, DAT, DRD1, DRD2, and ACTIN in striatal tissues of the indicated mouse groups. Protein expression levels were normalized to ACTIN (left) (*n* = 4). Representative images of DRD1 expression and quantification in the dorsal striatum of the indicated groups (*n* = 3–4). Scale bar, 200 µm (right). (B) Representative images and quantification of the marble burying test for indicated groups (*n* = 5–7) (left). Representative heat maps and social preference index of the three‐chamber social preference test (*n* = 5–7). "S" represents social stimulus and "NS" represents non‐social stimulus (right). (C) Representative images of cortical neurons expressing vG‐pH and MSNs expressing TdTomato in a cortex‐striatum mixed culture, along with quantification of mean response to 100 AP (left part). Scale bar, 5 µm. [*Shank3* +/+]_100AP_ = 0.22 ± 0.02 (n = 8); [*Shank3* −/−]_100AP_ = 0.16 ± 0.01 (*n* = 15), [*Shank3* +/+]_texo_  =  19.11 ± 2.46 s (*n*  =  5); [*Shank3* −/−]_texo_ = 33.75 ± 4.80 s (*n* = 5). Representative images and quantification of the mean value of 100 AP responses in the absence and presence of SKF‐82958 in *Shank3* −/− neurons (right part). Scale bar, 5 µm. [SKF‐82958 (−)]_100AP_ = 1 ± 0 (*n* = 9); [SKF‐82958 (+)]_100AP_ = 1.10 ± 0.03 (*n* = 9). (D) Western blot analysis and quantification of SHANK3, p‐ERK1/2, ERK1/2, p‐CREB, CREB, BDNF, p‐TrkB, TrkB, and ACTIN in striatum tissues of the indicated mouse groups. Protein expression levels were normalized to ACTIN (*n* = 3) (left and right top). mRNA levels of *Bdnf* transcripts derived from *Bdnf* exon1, *Bdnf* exone2, *Bdnf* exon4, and *Bdnf* exon6 for each indicated group (*n* = 6) (right bottom). Values are presented as means ± SEM. Statistical significance: ns, nonsignificant; *p < 0.05, **p < 0.01, ***p < 0.001. Two‐tailed unpaired Student's *t*‐test for (A and C, left); Two‐tailed paired Student's *t*‐test for (C, right); one‐way ANOVA with Dunnett's post hoc test for (B and D), compared to *Shank3* −/− + vehicle.

To further explore the relationship between behavioral phenotypes and synaptic function, we utilized a mixed‐culture system of co‐cultured cortical and striatal neurons derived from *Shank3* +/+ and *Shank3* −/− mice. These mice also express *TdTomato* under the control of the *Drd1a* promoter in the striatum, allowing for visualization of D1 MSNs (Figure 1C, left). Synaptic transmission was assessed using a pHluorin‐based assay (vGlut‐pHlourin [vG‐pH]), which provides a direct measure of synaptic activity. Following stimulation with 100 action potentials (APs), synaptic transmission was significantly reduced in *Shank3* −/− neurons (16 ± 1%) compared to *Shank3* +/+ neurons (22 ± 2%) (Figure 1C, left). Additionally, we investigated the rate of synaptic vesicle fusion during prolonged stimulation (2000 APs), revealing that *Shank3* −/− neurons exhibited a significantly slower fusion rate compared to *Shank3* +/+ neurons (∼1.8‐fold slower) (Figure 1C, left). We next sought to determine whether SKF‐82958 could restore synaptic function at cortico‐striatal synapses in *Shank3* −/− mice. Treatment with SKF‐82958 led to an approximately 10% increase in synaptic transmission at cortico‐striatal synapses (Figure 1C, right). These findings suggest that D1 receptor‐mediated synaptic dysfunction at cortico‐striatal synapses contributes to ASD‐like phenotypes, which can be ameliorated through the activation of the D1 receptor.

Biochemical analysis of the striatum region further revealed that *Shank3* −/− mice exhibited reduced ERK‐CREB signaling and BDNF expression, indicating D1 receptor signaling impairment in the striatum. Administration of SKF‐82958 restored D1 receptor‐mediated signaling and activated the BDNF‐TrkB signaling pathway, leading to upregulation of *Bdnf* mRNA expression (Figure 1D). These results demonstrate that SKF‐82958 effectively restores impaired D1 receptor signaling within the striatum of *Shank3* −/− mice.

However, several limitations remain. First, not all individuals with ASD or ASD animal models exhibit reduced dopamine signaling or diminished D1 receptor‐mediated signaling. Both hyper‐ and hypo‐dopaminergic states are associated with ASD‐like behaviors [2], suggesting that dopamine homeostasis is critical for behavioral regulation in ASD. Our study underscores the behavioral improvements observed when reduced dopamine signaling is restored to physiological levels through D1 receptor agonism. Second, the optimal clinical application of SKF‐82958 remains unclear. For potential clinical translation, a thorough evaluation of appropriate dosing regimens, administration frequency, and potential development of tolerance is required. To address the issue of tolerance, the D1 receptor allosteric modulators may represent a promising strategy [3]. Third, we observed an increase in D1 receptor‐mediated signaling within the striatum in *Shank3* −/− mice, as in a previous study [4]. However, we have newly found that in the cortico‐striatal projection context, the striatal D1 receptor signaling was downregulated. Further studies are needed to understand how this regional mismatch contributes to behavioral abnormalities. Finally, although SHANK3 and D1 receptors are enriched in the striatum, contributions from other brain regions cannot be excluded. Previous studies have demonstrated that region‐specific deletion of *Shank3* in other brain regions can contribute to behavioral abnormalities [5]. The potential effects in these additional brain subregions were not addressed in the present study and remain to be explored. Moreover, whether these phenomena are consistently reproduced in other *Shank3* mutant models has not yet been evaluated. Future studies involving conditional knockouts or patient‐derived point mutations are needed to enhance translational relevance.

In conclusion, *Shank3* −/− mice exhibit significant deficits in dopaminergic signaling within the striatum, particularly D1 receptor‐mediated cortico‐striatal projection. Furthermore, administration of the D1 receptor agonist SKF‐82958 restored social behaviors, synaptic function, and signaling deficits in *Shank3* −/− mice. Our study proposes a therapeutic strategy based on D1 receptor agonism for ASD cases characterized by reduced striatal D1 receptor signaling.

## Author Contributions

D.‐G.J. and S.H.K. supervised the whole project. D.‐G.J., S.H.K., J.‐S.K., and S.H. designed the research and wrote the manuscript. J.‐S.K. and H.S. performed the experiments, analyzed the data, and made the figures. M.A. and J.P. performed behavioral studies. M.A., Y.L., and J.L. maintained the animals. All authors have read and approved the final manuscript.

## Ethics Statement

All animal experiments were approved by Institutional Animal Care and Use Committees of Sungkyunkwan University (SKKUIACUC2020‐06‐36‐1, SKKUIACUC2021‐03‐09‐1, and KHSASP‐21‐060).

## Conflicts of Interest

The authors declare no conflicts of interest.

## Supporting information




**Supporting File 1**: mco270428‐sup‐0001‐SuppMat.docx

## Data Availability

The data presented in this study are available upon request to the corresponding author.

## References

[1] J. Peça , C. Feliciano , J. T. Ting , et al., “Shank3 Mutant Mice Display Autistic‐Like Behaviours and Striatal Dysfunction,” Nature 472, no. 7344 (2011): 437–442. 21423165 10.1038/nature09965 PMC3090611

[2] P. Kosillo and H. S. Bateup , “Dopaminergic Dysregulation in Syndromic Autism Spectrum Disorders: Insights from Genetic Mouse Models,” Front Neural Circuits 15 (2021): 700968. 34366796 10.3389/fncir.2021.700968 PMC8343025

[3] Y. Zhuang , B. Krumm , H. Zhang , et al., “Mechanism of Dopamine Binding and Allosteric Modulation of the Human D1 Dopamine Receptor,” Cell Research 31, no. 5 (2021): 593–596. 33750903 10.1038/s41422-021-00482-0 PMC8089099

[4] S. Tzanoulinou , S. Musardo , A. Contestabile , et al., “Inhibition of Trpv4 Rescues Circuit and Social Deficits Unmasked by Acute Inflammatory Response in a Shank3 Mouse Model of Autism,” Molecular Psychiatry 27, no. 4 (2022): 2080–2094. 35022531 10.1038/s41380-021-01427-0 PMC9126815

[5] A. L. Bey , X. Wang , H. Yan , et al., “Brain Region‐Specific Disruption of Shank3 in Mice Reveals a Dissociation for Cortical and Striatal Circuits in Autism‐Related Behaviors,” Translational Psychiatry 8, no. 1 (2018): 94. 29700290 10.1038/s41398-018-0142-6 PMC5919902

