# Supplementary material for: D1 Receptor Agonist Ameliorates Synaptic and Behavioral Deficits in a Shank3‐Deficient Mouse Model of Autism
Source: MedComm (2020). 2025 Oct 15;6(11):e70428. doi: 10.1002/mco2.70428 (PMC12521793; doi:10.1002/mco2.70428)
Supplement: Supplementary file 1 — Supporting File 1: mco270428‐sup‐0001‐SuppMat.docx [file MCO2-6-e70428-s001.docx]

**Supporting information**

**D1 receptor agonist ameliorates synaptic and behavioral deficits in a *Shank3*-deficient mouse model of autism**

Jun-Sik Kim^1^†, Sukmin Han^2^†, Mihyeon An^1^, Jinsu Park^1^, Yeongyeong Lee^1^, Jeein Lim^1^, Sung Hyun Kim^2*^, Dong-Gyu Jo^1*^

**Affiliations**

^1^ School of Pharmacy, Sungkyunkwan University, Suwon 16419, Korea.

^2^ Department of Neuroscience, Graduate School, Kyung Hee University, Seoul 02447, Korea

* Correspondence should be addressed to Dong-Gyu Jo (e-mail: [jodg@skku.edu](mailto:jodg@skku.edu)) and Sung Hyun Kim (e-mail: [sunghyunkim@khu.ac.kr](mailto:sunghyunkim@khu.ac.kr)).

† Jun-Sik Kim and Sukmin Han contributed equally.

**Content**

Materials and Methods (Pages 2-4)

References (Page 4)

**Materials and Methods**

**Animal studies** *Shank3* -/- (B6.129-*Shank3^tm2Gfng^*/J) mice and *Drd1a* promoter*-TdTomato* expressed transgenic mice (B6.Cg-Tg(Drd1a-TdTomato)6Calak/J) were obtained from the Jackson Laboratory (Bar Harbor, ME). Mice were housed in a specific pathogen-free facility under controlled temperature (22 ± 2 °C) and humidity (50–60 %) with a 12 h light/dark cycle, and had free access to standard chow diet and water. Genotyping was performed according to Jackson Laboratory’s (Bar Harbor, ME) protocol. For behavioral and biochemical analysis, we used male mice aged between 3 to 5 months. Littermate WT and *Shank3* -/- mice were generated by crossing *Shank3* +/- male and female mice. At 4 months, the mice were administered SKF-82958 (MedChemExpress, HA-10435A) with an IP injection of 2 mg/kg/day (Groups: *Shank3* +/+ + Vehicle, *Shank3* -/- + Vehicle, *Shank3* -/- + SKF-82958). Bean oil containing 2 % DMSO served as the vehicle for drug administration. One week after the initiation of injections, we commenced behavior studies (n = 5-7), and two weeks after the initial injection, the mice were sacrificed.

**Tissue sample preparation** All brain tissue samples were collected after the behavioral tests were completed. The mice were anesthetized with Zoletil (Virbac) and Rompun (Bayer). Following anesthesia, the mice were perfused with phosphate-buffered saline. For Western blotting analysis, the striatum was dissected from the brain, flash-frozen in liquid nitrogen, and stored at −80 °C until further use. For immunohistochemistry, samples were fixed with 4 % PFA at 4 °C overnight. The fixed tissues were then embedded in paraffin, and serial sections of 5 μm thickness were prepared.

**Western blot** Samples were lysed in RIPA buffer (Merck, 20-188) containing a phosphatase and protease inhibitor cocktail (Thermo Fisher, 78447). After incubating for 1 hour at 4 °C, the lysate was centrifuged at 10,000 g for 30 minutes at 4 °C to separate the supernatant from the pellet. The supernatant was collected for western blot analysis. Equal amounts of protein samples were loaded onto SDS-polyacrylamide gels and run until fully separated. The proteins were then transferred onto a 0.45 μm polyvinylidene difluoride membrane (Merck, M6250). The membranes were blocked with 5 % nonfat dry milk for 1 hour at room temperature (RT). After blocking, the membranes were incubated overnight at 4 °C with primary antibodies against ACTIN (Sigma-Aldrich, A5441), BDNF (Alomone, ANT-010), CREB (Cell Signalling, 9197S), DAT (Sigma-Aldrich, D6944), DRD1 (Abcam, ab81296), DRD2 (Merck, AB5084P), ERK1/2 (Cell Signalling, 9102S), p-CREB (Merck, 06-519), p-ERK1/2 (Cell Signalling, 9101S), p-TrkB (Merck, ABN1381), PSD95 (Cell Signalling, 3450), SHANK3 (Cell Signalling, 64555S), SYNAPTOPHYSIN (Zymed, 18-0130), TrkB (Cell Signalling, 4603S), and vGLUT1 (Merck, MAB5502). After washing, the membrane was incubated with a peroxidase-conjugated secondary antibody for 1 hour at RT. The immunoreactive bands were detected using an enhanced chemiluminescence solution (Cytiva, RPN2106).

**Immunohistochemistry (IHC)** Serially sectioned brain slices were deparaffinized and hydrated using a Histochoice clearing agent (VWR Life Science, 97060-934) and a series of ethanol concentrations. For antigen retrieval, the slices were incubated in Tris-EDTA buffer at 100 ℃ for 10 minutes. The slices were then blocked at RT for 1 hour in PBS containing Tween 20 and 3 % BSA (Bovogen, BSA100). After blocking, the DRD1 (Abcam, ab20066) primary antibody was applied, and the slices were incubated overnight at 4 ℃. The following day, the slices were washed and incubated with secondary fluorescent antibodies for 1 hour at RT. After a final wash, the slices were mounted using a mounting solution (Vector, H-1200-10). Images were captured with a fluorescence microscope (Leica, Thunder).

**Total RNA Isolation and Real-Time qPCR (RT-qPCR)** RNA was isolated according to the manufacturer’s instructions. Total RNA was extracted from striatum tissue samples using RNAiso plus solution (Takara, 9109) and total RNA extraction reagent. For complementary DNA (cDNA) synthesis, 500 ng of RNA from each sample was used with the PrimeScript RT Reagent kit with gDNA Eraser (Takara, PR047A) and CFX Connect (Bio-Rad, USA). PCR amplification was performed with the TB Green Premix Ex Taq II (Takara, PR820A). The PCR protocol included an initial denaturation at 95 ℃ for 30 seconds, followed by 40 cycles of 95 ℃ for 5 seconds, and 60 ℃ for 30 seconds. The differences between Ct values for the experimental and reference genes (*Hprt*) were calculated as ∆Ct. The primers used for real-time quantitative PCR were as follows: *Bdnf1* forward 5’-caagacacattaccttcctgcatct-3’, *Bdnf1* reverse 5’-accgaagtatgaaataaccatagtaag-3’, *Bdnf2* forward 5’-aagtgtttatcaccaggatctagccac-3’, *Bdnf2* reverse 5’-accgaagtatgaaataaccatagtaag-3’, *Bdnf4* forward 5’-tgtttactttgacaagtagtgactgaa-3’, *Bdnf4* reverse 5’-accgaagtatgaaataaccatagtaag-3’, *Bdnf6* forward 5’-gaagcgtgacaacaatgtgactc-3’, *Bdnf6* reverse 5’-accgaagtatgaaataaccatagtaag-3’, *Hprt* forward 5’-cctcctcagaccgcttttt-3’, and *Hprt* reverse 5’-aacctggttcatcatcgctaa-3’.

**Primary neurons culture** Primary cortical and striatal neurons were isolated from postnatal (0~2 day old) *Shank3* +/+ and -/- with *Drd1a* promoter*-TdTomato* expressed transgenic mice and plated on poly-ornithine coated coverslips. Neurons were transfected 7 days after plating and further incubated for 7–14 days in culture medium. All results are from at least three independent primary cultures.

**Optical imaging for synapse physiology (vG-pH assay)** For optical imaging of synaptic physiology, primary cultured neurons were transfected with CamKII promotor driven vGlut1-pHluorin plasmid (vG-pH) or Synaptophysin-pHluorin (Phy-pH) using the Ca^2+^ phosphate precipitation method.^1^ Briefly, vG-pH and Phy-pH were mixed with 2x HeBS (273 mM NaCl, 9.5 mM KCl, 1.4 mM Na2HPO4·PO2O, 15 mM D-glucose, 42 mM HEPES, pH 7.10) containing 2 mM Ca2+, after which the DNA mixture was applied to cortical and striatal neurons cultured for 7 days in vitro (DIV7). Cultured neurons at DIV14-21 transfected with either vG-pH or Phy-pH were used for imaging experiments. Neurons were applied with SKF-82958 (20 μM) for 10 min. Coverslips containing cultured neurons were positioned within a laminar-flow-perfused stimulation chamber on the stage of a custom-built, laser-illuminated epifluorescence microscope (Zeiss Observer). A back-illuminated EMCCD camera (Andor iXon Ultra 897; Model #DU-897U-CS0-BV) was employed to acquire live-cell images. A diode-pumped OBIS 488 laser (Coherent), shuttered by synchronizing the TTL on/off signal from the EMCCD camera during acquisition, was utilized as a light source. Fluorescence excitation/emission and collection were achieved using a 40 × Fluar Zeiss objective lens (1.3 NA) and 500–550 nm emission and 498 nm dichroic filters (Chroma). Action potentials (APs) were evoked by passing a 1-ms current pulse through platinum-iridium electrodes from an isolated current stimulator (World Precision Instruments). Neurons were perfused with Tyrode’s buffer consisting of 119 mM NaCl, 2.5 mM KCl, 2 mM CaCl2, 2 mM MgCl2, 25 mM HEPES, 30 mM glucose, 10 mM 6-cyano-7-nitroquinoxaline-2,3-dione (CNQX), and 50 mM D,L-2-amino-5-phosphonovaleric acid (AP5), adjusted to pH 7.4. All experiments were carried out at a controlled temperature of 30 °C. All images were acquired at 2 Hz with a 50-ms exposure.

**Image analysis** All images were analyzed using Image J (http://rsb.info.nih.gov/ij) with plugin Time Series Analyzer, which is available at https://imagej.nih.gov/ij/plugins/time-series.html. Synaptic boutons were selected as oval regions of interest (diameter, 10 pixels), and the intensity of fluorescence at synapses was measured. Fluorescence traces were analyzed using Origin Pro (ver. 2020).

**Three-chamber social preference test** The three-chamber social preference test was conducted as described previously.^2^ Briefly, the chamber with dimensions of 102 cm (length) x 47 cm (width) x 45 cm (height) was used for the test. On the first day, mice were habituated to the chamber for 10 min. On the following day, pre-tests were conducted for 10 min with non-social stimuli (Square wood blocks placed under cups). Next, the social preference tests were performed for 10 min with a non-social stimulus (A square wood block placed under a cup) and a social stimulus (An age- and sex-matched WT mouse under a cup). Mouse behavior was tracked using the video tracking system (Ethovision system, Noldus).

**Marble burying test** The marble burying test was performed as described in a previous study.^3^ Twenty marbles were placed as far apart as possible in a cage with bedding, and a mouse was placed in one corner of the cage and left undisturbed for 30 min. The number of buried marbles was then counted, with a marble considered buried if two-thirds of its surface area was covered by bedding.

**Statistical Analysis** All statistical analyses were conducted using GraphPad Prism 8.0 software, including two-tailed Student’s t-test and one-way ANOVA with Dunnett’s post-hoc tests, were performed using GraphPad Prism 8.0 software. Data are presented as mean ± standard error (SEM). Differences between groups were considered significant if P < 0.05, with significance levels denoted as *P < 0.05, **P < 0.01, and ***P < 0.001.

**References**

1. Bae JR, Lee W, Jo YO, et al. Distinct synaptic vesicle recycling in inhibitory nerve terminals is coordinated by SV2A. *Prog Neurobiol*. Nov 2020;194:101879.

2. Rein B, Ma K, Yan Z. A standardized social preference protocol for measuring social deficits in mouse models of autism. Nature Protocols. 2020;15(10):3464-3477.

3. Angoa-Pérez M, Kane MJ, Briggs DI, Francescutti DM, Kuhn DM. Marble burying and nestlet shredding as tests of repetitive, compulsive-like behaviors in mice. J Vis Exp. 2013;(82):50978.
